# Insects and Human Society: Ecological Services, Nutritional Value, Livelihood Support and Cultural Significance from Global Perspectives to Rural Venda, South Africa

**DOI:** 10.3390/insects17080812

**Published:** 2026-08-05

**Authors:** Khavhatondwi Rinah Mofokeng

**Affiliations:** Department of Life and Consumer Sciences, University of South Africa, P/Bag X6, Florida 1710, South Africa; enetshk@unisa.ac.za

**Keywords:** pollination, entomophagy, ecosystem services, indigenous ecological knowledge, edible insects, sustainable food systems, Venda, Limpopo, sub-Saharan Africa

## Abstract

Insects are fundamental to ecosystem functioning and human welfare, yet their multifaceted contributions to ecological services, food security, rural livelihoods, and cultural heritage remain insufficiently represented in mainstream scientific research and policy. Despite growing global recognition of insect decline and the potential of entomophagy to address food system challenges, integrated evidence linking global ecological knowledge with community-level indigenous practices is lacking, particularly in sub-Saharan African contexts. A narrative bibliometric review was conducted through systematic searches of Web of Science, Scopus, and PubMed databases, supplemented by FAO, IPBES, and MDPI Insects repositories. Searches were conducted between January 2024 and May 2026 using keyword combinations spanning entomophagy, ecosystem services, insect decline, indigenous knowledge and edible insects in Africa. Four thematic dimensions are synthesised: (1) ecological services, (2) nutritional value and food security, (3) economic contribution and livelihoods, and (4) socio-cultural and ethnomedicinal significance. The Venda region of Limpopo Province emerges as a compelling case study of how insect–human relationships intersect with biodiversity conservation, food security, and rural development. Actions include integrating indigenous knowledge with scientific research, strengthening sustainable community-based insect harvesting practices, and incorporating edible insects into South Africa’s food, nutrition, and biodiversity policies in alignment with global sustainability and biodiversity goals.

## Simple Summary

Insects are essential to human health and ecosystem sustainability, yet their contributions are often unnoticed in research, policy, and public discussion. This review evaluates the multiple roles that insects play in human societies, including their ecological functions, nutritional value, economic importance and cultural significance. Globally, insects provide critical ecosystem services such as pollination, nutrient cycling, decomposition, and natural pest regulation, while also serving as sustainable alternative protein sources. In sub-Saharan Africa, edible insects contribute to food security, household income, and traditional knowledge systems. Attention is given to the Venda communities of Limpopo Province, South Africa, where insects such as mopane worms are deeply embedded in local diets, livelihoods, and cultural practices. However, these benefits are increasingly threatened by habitat degradation, climate change, overexploitation, and the erosion of indigenous knowledge. This review highlights the importance of integrating indigenous ecological knowledge with scientific research and conservation efforts to promote sustainable insect use. The Venda region serves as a valuable example of how insect–human relationships can contribute to biodiversity conservation, rural development, and resilient food systems.

## 1. Introduction

Insects constitute the most species-rich and ecologically dominant class of animals on Earth, comprising an estimated 1.0 to 1.7 million described species and accounting for approximately 75–80% of all known animal diversity [1,2]. Beyond their taxonomic richness, insects perform roles in the functioning of terrestrial and aquatic ecosystems, providing services ranging from pollination and biological pest control to decomposition and soil formation [3,4]. Approximately 75% of the world’s leading food crops depend, at least in part, on insect-mediated pollination, representing an annual economic value estimated at over USD 235–577 billion [5]. The ecological importance of insects through food webs as prey for birds, reptiles, and mammals further underscores their centrality to biodiversity at every trophic level.

Despite these contributions, insect populations are under severe and accelerating pressure globally. Previous studies report declines of more than 75% in flying insect biomass in some protected areas of Europe over 27 years [6], and modelling studies project the extinction of 14–31% of insect species under moderate-to-high climate change scenarios by 2070 [7]. Habitat loss, agrochemical use, light pollution, invasive species, and climate change are identified as the primary drivers of these declines [8,9,10]. The ecological and socioeconomic consequences of losing insect biodiversity are profound and poorly quantified, particularly in the Global South where baseline data remain scarce.

Across sub-Saharan Africa, entomophagy is not a novel innovation but an ancient cultural practice, with over 500 edible insect species recorded in the region [11]. The mopane worm (*Gonimbrasia belina*), harvested extensively across the Limpopo Province in South Africa, alone generates an estimated USD 39–59 million in annual trade across southern Africa [12].

The Venda region, situated in the Vhembe District of Limpopo Province, South Africa, represents a particularly significant locale for understanding insect–human interactions. The Vhavenda people maintain a rich tradition of entomophagy and insect-based livelihood practices embedded within a broader system of indigenous ecological knowledge (IEK). Studies conducted in Vhembe municipalities stated that mopane worms are consumed by up to 94% of respondents in the region, with both nutritional and economic motivations reported [13]. The commercialisation of mopane worms in rural Limpopo has been reported as a poverty-alleviation mechanism, with individual households deriving seasonal livelihoods from this resource [14,15]. However, habitat degradation, urbanisation, and weak policy integration threaten the sustainability of these practices and the insect populations that support them [16].

This review addresses the multifunctional significance of insects in human society by synthesising evidence across four interlocking dimensions: (1) ecological services and biodiversity conservation; (2) nutritional value and food security; (3) economic contribution and livelihood support; and (4) socio-cultural and ethnomedicinal significance. The review progresses from global perspectives to African contexts, with particular attention to South Africa and the Venda region as a case study. The aim is to consolidate current knowledge, identify critical research gaps and articulate pathways for integrating indigenous knowledge with contemporary science to support sustainable insect utilisation, food system resilience, and rural development policy. While prior reviews have addressed edible insects, entomophagy in Africa, and ecosystem services of insects [3,4,12,17] independently, the present review offers a novel multi-scalar, integrative synthesis that bridges global ecological knowledge with subnational indigenous knowledge systems in a specific African community context. By centring the Venda region of South Africa as a detailed case study, this review advances beyond previous continental-level syntheses to demonstrate how insect–human relationships operate at the community level, connecting biodiversity conservation, food security, rural livelihoods, and cultural heritage within a single analytical framework. This place-based approach, informed by indigenous ecological knowledge, distinguishes the present review from the existing literature and responds to documented gaps in sub-Saharan African entomological scholarship.

## 2. Methodology

**Figure 1:**
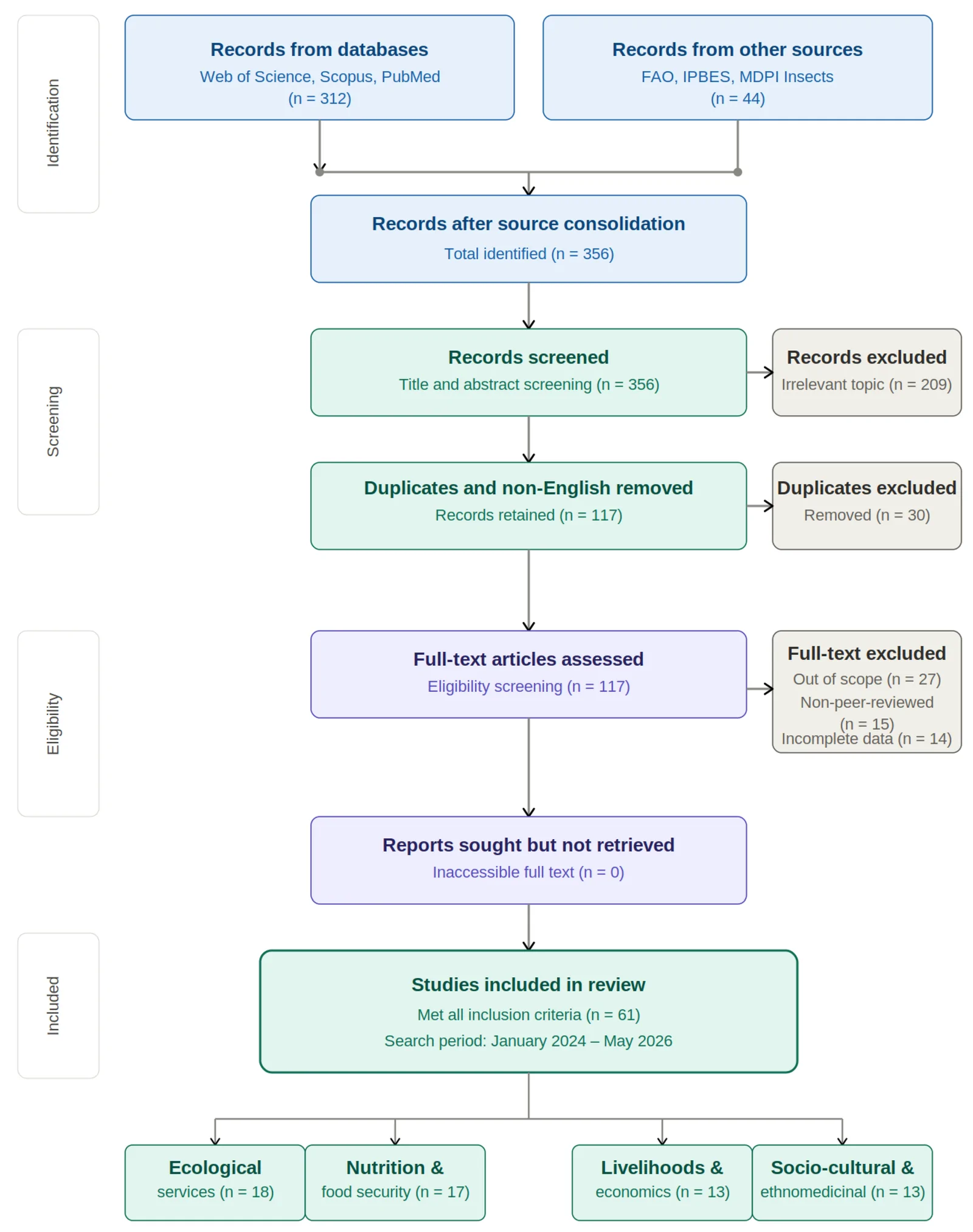
PRISMA 2020 flow diagram summarising the record identification, screening, eligibility, and inclusion process for the present review.

This review was conducted as a narrative synthesis of the peer-reviewed literature relevant to the multifunctional role of insects in human society. The search was conducted between January 2024 and May 2026, across Web of Science, Scopus, and PubMed databases, supplemented by FAO, IPBES, and MDPI Insects repositories. Inclusion criteria comprised peer-reviewed empirical studies, systematic reviews, and authoritative grey literature (FAO, IPBES reports) published from 2000 to 2026, in English, addressing insect ecological services, nutrition, livelihoods, socio-cultural significance, or conservation. Studies were excluded if they did not address insects specifically, lacked methodological transparency, or were outside the scope of the four thematic dimensions of the review. Records were managed and de-duplicated using [EndNote 21/Mendeley Reference Manager 2.x/Zotero 7.x] ([manufacturer, city, country]). Figure 1 provides a PRISMA-style workflow summarising the record identification, screening, eligibility, and inclusion process, generated using [Microsoft PowerPoint, Microsoft 365/the PRISMA2020 R package v1.1.2/Lucidchart]. A total of 61 sources met the inclusion criteria and are cited in this review.

The following keyword combinations were employed: entomophagy, edible insects, insect ecosystem services, insect pollination, biological pest control, insect decomposition, insect nutrition, indigenous knowledge insects Africa, mopane worm Limpopo, Venda edible insects, insect decline, insect conservation, sustainable food systems insects, insect livelihood, entomotherapy Africa, and insect cultural significance.

Published studies from 2000 to 2026 were prioritised, although foundational studies of earlier dates were included where directly relevant. Studies were selected based on relevance to the review objectives, methodological accuracy, and geographic scope. Qualitative synthesis was used to integrate findings across disciplines and geographic scales, from global analyses to community-level studies in the Vhembe District. The review structure progresses from macroscale ecological functions to mesoscale African perspectives, to microscale local Venda case studies, enabling multi-scalar analysis of insect–human interactions.

## 3. Results

### 3.1. Ecological Roles and Ecosystem Services of Insects

#### 3.1.1. Pollination Services

Pollination is among the most economically and ecologically significant services provided by insects. Bees (Hymenoptera: Apidae), flies (Diptera), beetles (Coleoptera), butterflies and moths (Lepidoptera), and wasps (Hymenoptera) are the principal insect pollinator groups globally [5,16]. An estimated 87.5% of all flowering plant species worldwide depend on animal pollination, with insects responsible for most of this service [5]. In agricultural systems, insect pollination contributes to the yield of approximately 75% of leading food crops, including many fruits, vegetables, and oilseeds [16]. The economic value of insect pollination services to global agriculture has been estimated at between USD 235 and 577 billion annually [5].

Honeybees (*Apis mellifera*) and stingless bees (Meliponini) are of cultural and economic relevance in sub-Saharan Africa, where bee products, including honey, propolis, and beeswax, support both subsistence and commercial livelihoods. In South Africa, the Cape bee (*Apis mellifera capensis*) and African honeybee (*Apis mellifera scutellata*) are ecologically important native pollinators, though their populations face ongoing pressure from pesticide exposure, habitat degradation, and Varroa mite infestations [5].

#### 3.1.2. Decomposition and Nutrient Cycling

Insects are major agents of organic matter decomposition and nutrient cycling in both terrestrial and aquatic ecosystems. Detritivores such as dung beetles (Scarabaeidae), termites (Isoptera), collembolans (Collembola), and various beetle and fly larvae facilitate the physical fragmentation and biochemical breakdown of dead plant material, animal carcasses, and faecal matter [3,4,18]. This decomposition accelerates nutrient mineralisation, increasing the availability of nitrogen, phosphorus, and other elements to plants, thereby driving primary productivity [3,4].

Termites are particularly important ecosystem engineers in African savannas and woodland ecosystems, including the mopane woodlands of Limpopo Province. Their subterranean activities improve soil structure, water infiltration, and organic matter incorporation [3]. Studies in restored forest ecosystems demonstrate that increases in decomposer insect diversity, including Collembolans and wood-decomposing beetles, significantly enhance biodegradation rates and soil health [3]. Dung beetles provide further services, including secondary seed dispersal, parasite suppression, and rapid dung removal that reduces methane emissions from livestock production systems [4].

In aquatic ecosystems, larval insects including mayflies (Ephemeroptera), stoneflies (Plecoptera), caddisflies (Trichoptera), and midges (Chironomidae) are central to the decomposition of leaf litter and the transfer of energy between riparian and aquatic food webs [8]. These groups also serve as bioindicators of freshwater ecosystem health, with their community structure reflecting water quality, pollution levels, and catchment disturbance [4]. The sensitivity of many aquatic insect orders to environmental change makes their monitoring critical for detecting ecosystem degradation.

#### 3.1.3. Biological Pest Regulation

Insects provide substantial regulating services through the predation and parasitism of agricultural and forest pests. *Parasitoid wasps* (Hymenoptera: Ichneumonidae, Braconidae), predatory beetles (Coleoptera: Carabidae, Coccinellidae), lacewings (Neuroptera), and predatory bugs (Hemiptera) collectively suppress pest populations in natural and managed ecosystems [3,4]. These services reduce crop losses and can partially substitute for chemical pesticide inputs in integrated pest management (IPM) systems [3,4].

The global economic value of insect-mediated pest regulation in agriculture is estimated at billions of dollars annually, though precise quantification remains methodologically challenging [3]. The disruption of these services through the widespread use of neonicotinoid and fipronil insecticides poses a significant risk: systemic pesticides affect non-target beneficial insects, including pollinators, parasitoids, and predators, creating feedback loops that can exacerbate pest outbreaks [9]. In sub-Saharan Africa, indigenous knowledge of beneficial insect roles in agroecosystems is often underutilised in formal agricultural extension services, representing a missed opportunity for sustainable pest management.

#### 3.1.4. Insect Decline: Global Trends and Drivers

Evidence of widespread and accelerating insect population decline has accumulated rapidly over the past two decades. A seminal study in Germany reported a decline of more than 75% in flying insect biomass in protected areas over 27 years [6]. Global assessments indicate that approximately 40% of insect species are declining worldwide, with more than 30% potentially threatened with extinction [8,19]. In tropical regions, where insect diversity is highest and documentation is most limited, the combined impacts of habitat loss, climate change, and agricultural intensification are considered particularly severe [20]. It should be emphasised that much of the evidence documenting insect population declines originates from studies conducted in temperate regions, particularly Europe and North America. In contrast, long-term monitoring data and population baselines for Afrotropical ecosystems, including the savanna and woodland landscapes of southern Africa, remain limited. This geographical imbalance creates significant knowledge gaps regarding the extent, drivers, and ecological consequences of insect population changes within African ecosystems. Consequently, while global trends suggest widespread declines in insect abundance and diversity, these findings should not be directly generalised to southern Africa without careful consideration of regional ecological, climatic, and land-use contexts. Further long-term studies and monitoring programmes are needed to generate sufficient evidence on insect population dynamics in African landscapes and to determine whether observed global patterns are reflected within these understudied regions [20].

The principal drivers of insect decline identified in the literature are: habitat loss and fragmentation due to agricultural expansion and urbanisation; agrochemical exposure including insecticides, herbicides and fungicides; climate change disrupting phenological synchrony between insects and their host plants or prey; light pollution affecting nocturnal insect behaviour and reproduction; invasive alien species and pathogen emergence [7,8,9]. These stressors interact synergistically, meaning that their combined effects often exceed the sum of individual impacts [8]. In high-intensity agricultural landscapes in tropical countries, insect abundance has been recorded at only 50% of levels in natural habitats [21].

For South Africa and the Vhembe region specifically, localised threats include mopane woodland degradation driven by overgrazing, charcoal production, and climate-driven shifts in tree distribution [22]. Climate projections suggest a potential contraction in the suitable range of *Gonimbrasia belina* under future warming scenarios, with direct consequences for the food security and livelihoods of communities dependent on this resource [22]. These threats highlight the urgency of integrating conservation planning with livelihood support strategies in rural Limpopo. A summary of the major ecosystem services discussed in this section is provided in Table 1.

### 3.2. Nutritional Value of Edible Insects and Food Security Implications

#### 3.2.1. Macronutrient and Micronutrient Composition

Edible insects are a nutritionally rich food source, characterised by high protein content, beneficial fatty acid profiles, and important micronutrients including iron, zinc, calcium, and B-vitamins [10,11]. Protein content varies substantially across orders: Lepidoptera (caterpillars) contain 20–80% protein on a dry weight basis, Coleoptera (beetles) provide rich carbohydrate profiles (7–54% dry weight), and Lepidoptera also exhibit the highest fat content (10–50% dry weight) [11]. The mopane worm (*Gonimbrasia belina*), among the most nutritionally studied edible insects in southern Africa, contains approximately 48–72% protein and 10–15% fat on a dry weight basis, with an amino acid profile broadly comparable to conventional animal proteins [23,24].

Iron content is a particularly important nutritional attribute of edible insects in the African context, where iron-deficiency anaemia is prevalent. Locusts, for instance, contain 8–20 mg iron per 100 g dry weight, compared to approximately 6 mg per 100 g for beef [10]. Zinc, which is essential for immune function and child development, is present in nutritionally significant concentrations in mopane worms and termites [10]. Several insect species also provide dietary fibre in the form of chitin in their exoskeletons, which may support gut microbiome health and immune function, though bioavailability and digestibility remain areas requiring further research [13].

The nutritional composition of edible insects is influenced by species, developmental stage, diet, and processing methods. Drying and roasting are the predominant processing methods in southern African communities, including Vhembe, which affect fatty acid profiles and water-soluble vitamin retention [11]. Post-harvest management practices among Venda communities, including sun-drying and dry-roasting of mopane worms, are effective at extending shelf life and reducing microbial load, though standardisation and cold chain development would enhance commercial scalability [14]. It is important to acknowledge that the nutritional composition of edible insects can vary considerably depending on species, developmental stage, feeding substrate, environmental conditions, and processing methods. Furthermore, reported nutrient values are often generated using different analytical techniques and laboratory protocols, which can contribute to inconsistencies across studies. Consequently, the nutritional ranges presented in this review and in Table 2 should be interpreted as indicative estimates rather than definitive values. In addition, the reported macronutrient and micronutrient contents do not necessarily reflect the proportion of nutrients that can be absorbed and utilised by the human body. Factors such as nutrient bioavailability, digestibility, the presence of antinutritional compounds, food preparation methods, and individual physiological differences can significantly influence the nutritional benefits ultimately derived from edible insect consumption. Therefore, caution should be exercised when comparing nutrient profiles across studies or when using gross composition data to infer nutritional outcomes for human populations [11]. The nutritional composition of selected edible insect species compared to beef is summarised in Table 2.

#### 3.2.2. Environmental Sustainability of Insect-Based Protein

The environmental credentials of insect production compared to conventional livestock are well documented and represent a key driver of global interest in entomophagy. Insects require substantially less land and water per kilogram of protein produced: insect farming requires between 50% and 90% less land than equivalent conventional livestock production [26]. Feed conversion efficiency is significantly higher in insects; crickets, for example, require approximately 1.7 kg of feed to produce 1 kg of body mass, compared to 8 kg of feed for 1 kg of beef [27]. Greenhouse gas emissions from insect production are markedly lower than from ruminant livestock, with insects producing far less methane per unit of biomass; crickets produce approximately 80 times less methane per kilogram than cattle [26].

Insect farming also presents opportunities for the valorising of organic waste streams through bioconversion, supporting circular economy principles. Black soldier fly larvae (*Hermetia illucens*) are particularly effective bioconversion organisms, transforming organic waste into high-quality protein biomass while significantly reducing waste volume [28]. These characteristics position insect production as a promising component of future sustainable food systems, particularly given that global protein demand is projected to increase by approximately 60% by 2050, in line with population growth and dietary transitions [29]. However, critical perspectives note that consumer acceptance barriers, particularly in Western markets, and limited investment relative to plant-based alternatives constrain the near-term scalability of edible insect industries as a substitute for conventional meat [30]. Furthermore, the favourable land-use, water-use, and greenhouse gas estimates reported in the literature are primarily based on life-cycle assessments of controlled, industrial-scale insect farming systems. These findings may not accurately reflect the environmental performance of wild harvesting practices, which remain the predominant source of edible insects in many parts of sub-Saharan Africa. In addition, comparisons on a per-kilogram basis are influenced by methodologies such as system boundaries and co-product allocation approaches, making direct comparisons with conventional livestock production challenging [26]. Key environmental sustainability metrics comparing insect and conventional livestock production are presented in Table 3.

#### 3.2.3. Edible Insects and Food Security in Sub-Saharan Africa

In sub-Saharan Africa, approximately 500 edible insect species are recorded, representing a significant proportion of the estimated 2100 edible species globally [11,12]. Central Africa supports the highest species richness for consumption (256 species), followed by southern Africa (164 species), eastern Africa (100 species), and western Africa (91 species) [12]. Most of these species are harvested from the wild rather than farmed, and their consumption is deeply integrated into subsistence food systems, particularly in rural communities where access to formal protein markets is limited [11,17,31].

Edible insects contribute to the four pillars of food security: availability (seasonal abundance of wild-harvested species), access (low-cost procurement relative to conventional meat), utilisation (high nutritional value), and stability (supplementary protein during periods of staple food scarcity) [10,14]. Studies conducted in rural Madagascar demonstrated that insect consumption contributes significantly to animal protein intake [32], particularly during the humid season when other protein sources are scarce, and that insects are consumed across socioeconomic strata without significant differential by income level [32]. This equity dimension of entomophagy is an important feature for policy consideration.

The FAO’s framing of edible insects within Sustainable Development Goal 2 (Zero Hunger) has elevated entomophagy in international food policy discourse [25]. In South Africa, the National Food and Nutrition Security Policy framework does not formally integrate edible insects as a dietary resource despite their established contribution to nutritional outcomes in Limpopo communities [14]. This policy gap constrains support for sustainable harvesting infrastructure, processing standardisation, and market development for insect-based foods. It is important to note that much of the existing evidence on the relationship between entomophagy and food security is derived from cross-sectional studies and self-reported dietary consumption data, which are inherently limited in their ability to establish causality. While numerous studies report positive associations between edible insect consumption, dietary diversity, protein intake, and household food security, these findings often reflect correlations rather than direct cause-and-effect relationships. Furthermore, self-reported consumption data may be affected by recall bias, seasonal variations in insect availability, and differences in cultural practices, which can influence the accuracy and comparability of findings across studies.

In many rural communities, edible insects are consumed alongside a range of other food sources and livelihood strategies, making it difficult to isolate their specific contribution to nutritional outcomes and food security. Factors such as household income, agricultural production, market access, education, and broader socioeconomic conditions may also influence both insect consumption and food security status. As a result, the extent to which edible insects directly improve nutritional status, reduce malnutrition, or enhance household resilience remains insufficiently understood [14]. To address these knowledge gaps, there is a need for longitudinal studies, controlled intervention trials, and quantitative assessments that track changes in dietary intake, nutritional indicators, and household food security over time. Such research would provide stronger evidence regarding the long-term role of edible insects in improving food and nutrition security and would help inform policies and programmes aimed at integrating entomophagy into sustainable food systems [14].

### 3.3. Livelihood Support and Economic Contributions

#### 3.3.1. Insect Trading and Rural Economies in Sub-Saharan Africa

Across sub-Saharan Africa, the harvesting, processing, and sale of edible insects form an important component of the informal economy and provide a vital source of seasonal income for many rural households. For communities with limited access to formal employment opportunities, edible insect trading serves as a livelihood diversification strategy that supports household resilience, particularly during periods of food scarcity and economic hardship. Among the most economically significant species is the mopane worm (*Gonimbrasia belina*), whose trade is estimated to generate between USD 39 and 59 million annually across southern Africa [12]. The increasing commercialisation of edible insects has expanded market opportunities beyond local consumption, creating value chains that connect rural harvesters, traders, processors, and retailers to regional and urban markets. This growing demand not only contributes to household incomes but also creates employment opportunities, particularly for women and youth who are actively involved in harvesting, processing, packaging, and marketing activities. However, the sustainability and long-term economic benefits of this sector depend on effective resource management, value addition, market development, and the implementation of policies that support equitable participation throughout the edible insect value chain [15].

In the Vhembe District of Limpopo Province, five insect groups belonging to four orders are regularly traded in informal markets: mopane worms (*Gonimbrasia belina*, 42% of trade volume), termites (soldiers, workers, and alates), and Gynanisa caterpillars [14].

Mopane worms have also entered formal retail markets in South Africa, with Spar stores in Vhembe District now stocking *Gonimbrasia belina* and *Cirina forda* at prices ranging from R19 to R170 per kilogram [33], signalling a gradual formalisation of the edible insect value chain.

#### 3.3.2. Insect Farming as an Economic Development Opportunity

While wild harvesting remains the dominant mode of insect acquisition in sub-Saharan Africa, growing recognition of the economic potential of insect farming particularly for feed ingredients and human food is attracting investment and policy attention globally. The global edible insect market was valued at approximately USD 400 million in 2018 and projected to exceed USD 1.2 billion by 2023 [24]. In the Asia–Pacific region alone, the market is projected to exceed USD 270 million [24]. Commercial insect farming operations, primarily producing black soldier flies, mealworms, and crickets, have expanded rapidly in Europe, North America, and Asia, processing waste streams into protein biomass for aquaculture and livestock feed [3].

In South Africa, the insect farming sector remains nascent but is receiving increasing attention from agricultural development agencies and private sector actors. The potential for small-scale insect farming enterprises to supplement wild-harvested supplies, reduce overharvesting pressure on wild populations, and provide year-round income stability for rural women is significant but under-explored in formal policy frameworks [14]. Key barriers include lack of processing infrastructure, limited access to finance, absence of food safety regulations specific to edible insects, and inadequate technical support for scaling artisanal production methods [14].

Only four of 36 government policy documents reviewed in a study of economic development strategies in Limpopo Province explicitly referenced edible insects, and only the mopane worm was included in any formal planning document [14]. This represents a significant policy gap given the scale of the existing informal insect economy and its role in poverty alleviation. Effective policy integration would require coordinated action across food safety (regulation of edible insects under South African food law), agriculture (extension support for semi-domestication and sustainable harvesting), trade (market development and value chain formalisation), and environmental management (habitat conservation for host tree species) [14].

### 3.4. Socio-Cultural and Ethnomedicinal Significance

#### 3.4.1. Cultural Dimensions of Entomophagy in Africa

Insects occupy a rich and varied cultural space in African societies, extending well beyond their roles as food commodities. Across sub-Saharan Africa, insects feature prominently in cultural narratives, ritual practices, symbolic systems, and intergenerational knowledge transmission [34,35]. Van Huis [34] documented the cultural significance of locusts, grasshoppers, and crickets in sub-Saharan Africa, encompassing their role as metaphors in oral literature, their use in ceremonies, and their significance as seasonal markers in agricultural and ecological calendars. In many societies, the arrival of specific edible insect species signals seasonal transitions and informs planting or harvesting decisions.

Among the Vhavenda people of Limpopo Province, the harvesting and preparation of mopane worms is embedded in social practices that affirm community bonds, transmit ecological knowledge across generations, and assert cultural identity in the face of modernisation pressures [13,36]. Women’s role as the primary harvesters and processors of mopane worms in Vhembe carries social significance beyond economic function, conferring status and preserving gendered knowledge systems [14]. The decline in entomophagy observed in KwaZulu-Natal, attributed to Western dietary acculturation and the social stigma associated with insect eating in urbanised settings [13], highlights the cultural vulnerability of these practices and the importance of cultural affirmation in conservation and food security interventions.

The concept of entomophagy acceptance or ‘insect neophobia’ in Western consumer contexts contrasts markedly with the cultural embeddedness of insect consumption in African communities [3]. Consumer behaviour research consistently finds that cultural familiarity and positive dietary experiences in childhood are the strongest predictors of insect food acceptance, reinforcing the importance of preserving and valorising existing entomophagous food cultures rather than constructing insect consumption as novel behaviour [3].

#### 3.4.2. Ethnomedicinal Applications and Entomotherapy

The medicinal use of insects (entomotherapy) is documented across sub-Saharan Africa and constitutes an important dimension of traditional healing systems. A comprehensive systematic review of invertebrate use in African traditional medicine identified 119 invertebrate species from 40 orders used to treat 191 symptoms across 20 sub-Saharan African countries [37]. Insects were the most frequently used invertebrate group in traditional medicine, with *Apis mellifera* (honeybees) cited for treating 35% of documented symptoms, including wounds, arthritis, cough, stomach ailments, and fever [37]. Termites, ants, beetles, and Orthoptera were among the other commonly cited medicinal insect groups.

In Burkina Faso, a study of 60 traditional healers across two phytogeographical zones documented 19 medicinal insect species from six orders, used in the treatment of at least 78 pathologies [38]. Hymenoptera, particularly Apis mellifera and various wasp and ant species, accounted for the highest frequency of citation. Honey, bee venom, propolis, and royal jelly are among the most empirically studied insect-derived medicinal substances, with documented antimicrobial, anti-inflammatory, and wound-healing properties [39]. In South Africa, the indigenous use of bee products in traditional medicine (muti) is widespread, though systematic documentation of Venda-specific entomotherapy practices remains limited in the published literature.

The intersection of entomotherapy and biodiversity conservation is a critically under-researched domain. As wild insect populations decline and indigenous knowledge erodes through urbanisation and cultural change, the medicinal knowledge embedded in entomotherapy practices is at risk of irreversible loss [37,40]. Ethnoentomological documentation projects, particularly those conducted in collaboration with traditional healers and community knowledge holders in regions such as the Vhembe District, represent an urgent conservation priority [40].

### 3.5. The Venda Region as a Case Study: Localised Insect–Human Interactions

#### 3.5.1. Ecological and Socioeconomic Context

The Vhembe District Municipality, encompassing the historic Venda homeland in the far north of Limpopo Province, South Africa, borders Zimbabwe to the north, Botswana to the west, and Mozambique to the northeast. It is one of the most biodiversity-rich yet economically marginalised regions in South Africa, characterised by high unemployment, heavy reliance on government grants, and a rural economy with limited industrial diversification [14]. The district encompasses significant areas of mopane woodland (*Colophospermum mopane*), which are the primary habitat for *Gonimbrasia belina* and support multiple edible insect species of commercial and nutritional importance [22,23].

The Vhavenda people have inhabited the region for centuries and maintain a sophisticated system of ecological knowledge governing seasonal resource use, including the harvesting of edible insects. This knowledge encompasses the phenology of insect emergence, appropriate harvesting techniques to protect future populations, processing methods that enhance safety and palatability, and trade networks linking village harvesters to regional markets [13,36]. The region has been the subject of a growing body of entomological and social science research, making it one of the best-documented localities for edible insect practices in southern Africa.

#### 3.5.2. Edible Insect Species and Harvesting Practices

In the Vhembe District, mopane worms (*Gonimbrasia belina*) dominate both consumption and trade, being preferred by 94% of respondents in surveys of insect consumption in four local municipalities [13]. Other species consumed and traded include termite alates (*Macrotermes natalensis* and related species), Gynanisa caterpillars, grasshoppers (Acrididae), and stinkbugs (*Encosternum delegorguei*, *halyomorpha*). The primary edible insect species consumed and traded in the Vhembe District are listed in Table 4.

Harvesting of mopane worms in Vhembe involves community-regulated practices that have evolved to manage the resource sustainably over generations, including selective harvesting of mature larvae, leaving pupae to complete their life cycle, and respecting communal harvesting norms across property regimes [41]. Studies across nine sites in Vhembe and Mopani districts found that governance of mopane worm harvesting operates under a complex mosaic of private, public, and communal property rights, with informal community rules often more effective than formal regulations at managing harvesting pressure [41]. However, the commercialisation of mopane worms, driven by expanding urban markets and export demand, is straining traditional governance systems and contributing to overexploitation in some areas [41].

#### 3.5.3. Threats and Conservation Challenges

The sustainability of edible insect resources in the Vhembe District faces several converging threats. Habitat degradation of mopane woodlands driven by overgrazing, firewood harvesting, charcoal production, and agricultural encroachment directly reduces the carrying capacity for mopane worms and associated insect communities [22]. Climate change is projected to cause range contractions in *Colophospermum mopane* under warming and drying scenarios, with downstream consequences for the distribution and abundance of *Gonimbrasia belina* [22]. Overexploitation of mopane worm populations through unsustainable harvesting intensities, exacerbated by commercial pressure and weakened community governance, represents an additional stressor [41].

Urbanisation and Western dietary acculturation are eroding the intergenerational transmission of indigenous insect knowledge in the region, with younger generations in peri-urban areas showing reduced engagement with traditional harvesting and processing practices [13]. This knowledge erosion is compounded by the lack of formal documentation of indigenous ecological knowledge related to insects in Vhembe, creating risks of irreversible cultural and biological loss [40]. Paradoxically, the very communities with the deepest knowledge of insect ecology and utilisation receive the least support from formal research and extension systems.

#### 3.5.4. Opportunities for Sustainable Insect Utilisation in Venda

Despite these challenges, the Vhembe region presents significant opportunities for developing integrated, community-centred approaches to sustainable insect utilisation. The intersection of rich indigenous knowledge, established market demand, biodiversity assets, and high social need creates conditions amenable to multi-stakeholder interventions [14]. Priority areas for intervention include: (1) participatory documentation and formalisation of indigenous insect harvesting knowledge; (2) development of sustainable harvesting guidelines co-produced with traditional governance structures; (3) investment in processing infrastructure (drying, packaging, storage) to reduce post-harvest losses and improve product quality; (4) integration of edible insects into formal food safety regulation and nutrition policy; and (5) habitat conservation programmes targeting mopane woodland restoration in harvesting areas.

The partial entry of mopane worms and *Cirina forda* into formal retail chains through Spar stores in Vhembe represents a potential inflection point for value chain development, offering harvesters access to higher-margin markets while raising product safety standards [33]. However, such formalisation must be carried out to ensure that benefits are equitably distributed and that the increased commercial pressure does not exacerbate overharvesting, by linking value chain development with conservation incentives; for example, payments for ecosystem services linked to mopane woodland offers a potentially transformative policy instrument for the region.

### 3.6. Insect Conservation: Integrating Ecological and Indigenous Knowledge Perspectives

The convergence of global insect decline, growing appreciation of insects’ ecological and socioeconomic value, and the erosion of indigenous insect knowledge creates a compelling case for integrated conservation approaches that bridge Western science and traditional ecological knowledge. Effective insect conservation requires action at multiple scales: habitat protection at the landscape scale; reduction of pesticide loading in agricultural landscapes; light pollution mitigation in and around urban areas; climate change mitigation; and community-level governance of insect harvesting [7,8,9].

In the African context, traditional resource governance systems, including seasonal harvesting bans, communal access rules, and sacred site protections, historically provided important ecological buffers that prevented the overexploitation of insect resources [41]. These systems are eroding under pressures of commercialisation, formal land tenure changes, and governance failures, necessitating deliberate efforts to document, strengthen, and formally recognise indigenous governance mechanisms in national and provincial conservation frameworks [40,41].

The integration of indigenous ecological knowledge (IEK) with contemporary entomological science holds promise for the Venda region and analogous contexts across sub-Saharan Africa. IEK often captures longitudinal ecological observations spanning decades of phenological change, population dynamics, and habitat associations that formal monitoring programmes cannot replicate [40]. Community-based monitoring programmes that train local harvesters as ecological observers, coupled with formal analytical frameworks, represent an approach that simultaneously advances scientific knowledge and empowers indigenous communities as knowledge producers rather than research subjects. It should be acknowledged, however, that tensions exist between portrayals of IEK as inherently sustainable and empirical evidence showing that traditional governance systems can fail under commercialisation pressures, as documented in the Vhembe mopane worm case [41]. Moreover, the literature on edible insects and conservation is predominantly descriptive and cross-sectional; longitudinal studies capable of attributing population trends to specific governance or land-use drivers remain scarce. Future syntheses should evaluate the quality of evidence rather than treating all findings as equally robust.

South Africa’s National Biodiversity Strategy and Action Plan (NBSAP) and the associated Biodiversity Act (No. 10 of 2004) provide a legal framework within which insect conservation could be strengthened, but insects receive minimal explicit attention in current biodiversity policy instruments. Advocating for the inclusion of insects including edible species—in monitoring, assessment, and conservation programmes under South Africa’s national biodiversity infrastructure—represents an important policy priority [14,42].

### 3.7. Research Gaps and Future Directions

Notwithstanding the substantial body of research reviewed here, significant knowledge gaps constrain evidence-based policy and practice for insect utilisation and conservation. The following priority research areas are identified and summarised in Table 5.

Baseline biodiversity data on insect communities in southern African savanna and woodland ecosystems, including the Vhembe District, are severely limited. Long-term population monitoring programmes are needed to detect trends in abundance and diversity of both ecologically functional and edible insect species and to attribute observed changes to specific drivers [8].

The nutritional bioavailability of micronutrients in edible insect species consumed in southern Africa requires more rigorous investigation. While gross nutritional composition data are reasonably well documented for key species including mopane worms, human feeding studies examining iron and zinc bioavailability from insect sources are scarce [11].

The economics and governance of the informal edible insect trade in Limpopo Province require comprehensive mapping to inform value chain development and policy responses. Existing studies provide important insights [14,33], but are primarily cross-sectional; longitudinal studies tracking the dynamics of market integration, benefit distribution, and harvesting pressure over time would provide critical evidence for policy design.

Systematic ethnoentomological documentation of the Vhavenda people’s insect knowledge, including species-specific ecological knowledge, harvesting calendars, processing methods, and medicinal uses, is urgently needed before this knowledge is further eroded by generational change. Collaborative, community-led documentation projects that apply the principles of free, prior, and informed consent and ensure benefit-sharing with knowledge holders represent the appropriate methodological approach [40].

Climate change modelling of the distribution and abundance of key edible insect species in Limpopo Province, including *G. belina* and *M. natalensis*, is needed at finer spatial resolution than currently available, to inform adaptive management and community livelihood planning [22].

## 4. Conclusions

This review has demonstrated the profound and multifaceted roles of insects in human society, from their foundational ecological functions to their nutritional contributions, livelihood support, and deep cultural significance. Insects are not peripheral to human welfare but central to it, maintaining the ecosystem services on which food production and biodiversity conservation depend, providing nutritionally rich food resources for millions of people, supporting rural economies across sub-Saharan Africa, and carrying cultural meanings that bind communities to their ecological landscapes.

The Venda region of Limpopo Province, South Africa, emerges from this review as a compelling case study of the intersection between insect ecology, indigenous knowledge, food security, and rural development. The Vhavenda people’s traditions of mopane worm harvesting and insect-based livelihoods represent a sophisticated, place-based adaptation to local ecological conditions that has sustained communities for generations. These traditions are now under pressure from habitat degradation, climate change, commercialisation, and cultural change, and require urgent attention from researchers, policymakers, and conservation practitioners. The path forward requires integrating indigenous ecological knowledge with contemporary science, formalising insect resources within food safety and nutritional policy frameworks, strengthening community governance of insect harvesting, investing in habitat conservation for host plant species, and supporting value chain development that benefits harvesters equitably. These actions are not separate from but deeply interconnected with global commitments to biodiversity conservation, sustainable food systems and rural poverty reduction under the Sustainable Development Goals and the Kunming–Montreal Global Biodiversity Framework. Insects, in all their ecological and cultural richness, must be placed at the centre of these efforts.

## Figures and Tables

**Table 1:** Major ecosystem services provided by insects and their ecological/economic significance.

Ecosystem Services	Key Insect Groups	Ecological/Economic Significance	Primary References
Pollination	Bees, flies, beetles, butterflies, wasps	Supports 75% of leading food crops; valued at USD 235–577 billion/year globally	[5,16]
Decomposition and Nutrient Cycling	Dung beetles, termites, collembolans, fly larvae	Accelerates nutrient mineralisation; improves soil structure and water infiltration	[3,4]
Biological Pest Regulation	*Parasitoid wasps*, predatory beetles, lacewings, predatory bugs	Suppresses agricultural and forest pests; reduces chemical pesticide inputs	[3,4]
Bioindicator/Water Quality	Mayflies, stoneflies, caddisflies, midges	Community structure reflects water quality, pollution and catchment disturbance	[3,4]
Food Web Support	Most insect orders (larvae and adults)	Prey for birds, reptiles, and mammals; central to biodiversity at every trophic level	[1,2,3]
Seed Dispersal	Dung beetles	Secondary seed dispersal and parasite suppression in livestock systems	[4]

**Table 2:** Approximate nutritional composition of selected edible insect species compared to beef.

Species (Common Name)	Order	Protein (% DW)	Fat (% DW)	Iron (mg/100 g DW)	Zinc	Reference
*Gonimbrasia belina* (Mopane worm)	Lepidoptera	48–72	10–15	High	Yes	[11,23,24]
*Locusta migratoria* (Locust)	Orthoptera	60–75	5–20	8–20	Moderate	[3,11]
*Macrotermes natalensis* (Termite alate)	Isoptera	35–45	30–45	Moderate	Yes	[10,11]
*Gryllus bimaculatus* (Cricket)	Orthoptera	55–70	10–30	Moderate	Yes	[10,24]
*Encosternum delegorguei* (Stinkbug)	Hemiptera	~50	~35	Moderate	Present	[14]
*Hermetia illucens* (Black soldier fly larva)	Diptera	40–44	29–35	Low–Mod.	Present	[3,25]
Beef (reference livestock)	—	~26	~15	~6	Present	[10]

DW = dry weight. Values are approximate ranges compiled from the cited literature. ‘High’/‘Moderate’/’Present’ denote qualitative assessments where precise data were unavailable.

**Table 3:** Environmental sustainability comparison between insect farming (crickets) and conventional beef cattle production.

Environmental Indicator	Insects (Crickets)	Beef Cattle	Source
Feed conversion ratio (kg feed/kg body mass)	~1.7	~8	[27]
Land use relative to beef	10–50% of beef land	100% (baseline)	[26]
Greenhouse gas (methane) emissions	~80× less than cattle/kg	Baseline	[26]
Water use	Substantially lower	High	[26]
Waste valorisation capacity	High (organic bioconversion)	Limited	[28]
Projected protein demand coverage by 2050	Significant contribution possible	Constrained by resource limits	[29]

**Table 4:** Edible insect species consumed and traded in the Vhembe District, Limpopo Province, South Africa.

Species	Order	Common Name	Peak Season	Primary Use
*Gonimbrasia belina*	Lepidoptera	Mopane worm	November–January; April–May	Food, trade (42% of trade volume)
*Macrotermes natalensis* (alates)	Isoptera	Termite alate	First summer rains	Food, trade
*Gynanisa* spp.	Lepidoptera	Gynanisa caterpillar	Seasonal (summer)	Food, trade
*Acrididae* spp.	Orthoptera	Grasshopper	Opportunistic	Food, trade
*Encosternum delegorguei*	Hemiptera	Stinkbug	Summer	Food, trade
*Cirina forda*	Lepidoptera	Pallid emperor moth larva	Seasonal	Food, formal retail (Spar)

*Sources*: [13,14,33]. Trade volume proportion for mopane worm from [14].

**Table 5:** Summary of priority research gaps and recommended future directions.

#	Research Gap	Recommended Action	Policy/Sector Relevance
1	Baseline insect biodiversity data in Vhembe and southern African woodland ecosystems	Establish long-term insect population monitoring programmes	Biodiversity conservation; NBSAP implementation [8]
2	Nutritional bioavailability of micronutrients from edible insects	Conduct human feeding trials for iron and zinc bioavailability	Food security; nutrition policy; SDG 2 [11,27]
3	Economics and governance of informal insect trade in Limpopo	Commission longitudinal value chain and governance studies	Rural development; trade policy; poverty alleviation [14,33]
4	Systematic ethnoentomological documentation of Vhavenda insect knowledge	Community-led documentation with FPIC and benefit-sharing provisions	IEK integration; cultural heritage; conservation [40]
5	Climate change impacts on distribution of *G. belina* and *M. natalensis*	High-resolution species distribution modelling under climate scenarios	Adaptive management; food security planning [22]

## Data Availability

No new data were created or analysed in this study. Data sharing is not applicable to this article.
